# Supplementary material for: Thermal contrast enhancement predicts paradoxical heat sensation
Source: Commun Psychol. 2024 Apr 24;2:37. doi: 10.1038/s44271-024-00083-8 (PMC11332243; doi:10.1038/s44271-024-00083-8)
Supplement: Supplementary file 3 — Reporting Summary [file 44271_2024_83_MOESM3_ESM.pdf]

nature portfolio

Corresponding author(s): Alexandra G. Mitchell & Francesca Fardo

Last updated by author(s): 31.01.2024

Reporting Summary

Nature Portfolio wishes to improve the reproducibility of the work that we publish. This form provides structure for consistency and transparency in reporting. For further information on Nature Portfolio policies, see our Editorial Policies and the Editorial Policy Checklist.

Statistics

For all statistical analyses, confirm that the following items are present in the figure legend, table legend, main text, or Methods section.

n/a	Confirmed	
☐	☒	The exact sample size (*n*) for each experimental group/condition, given as a discrete number and unit of measurement
☐	☒	A statement on whether measurements were taken from distinct samples or whether the same sample was measured repeatedly
☐	☒	The statistical test(s) used AND whether they are one- or two-sided<br>*Only common tests should be described solely by name; describe more complex techniques in the Methods section.*
☐	☒	A description of all covariates tested
☐	☒	A description of any assumptions or corrections, such as tests of normality and adjustment for multiple comparisons
☐	☒	A full description of the statistical parameters including central tendency (e.g. means) or other basic estimates (e.g. regression coefficient) AND variation (e.g. standard deviation) or associated estimates of uncertainty (e.g. confidence intervals)
☐	☒	For null hypothesis testing, the test statistic (e.g. *F*, *t*, *r*) with confidence intervals, effect sizes, degrees of freedom and *P* value noted<br>*Give P values as exact values whenever suitable.*
☒	☐	For Bayesian analysis, information on the choice of priors and Markov chain Monte Carlo settings
☒	☐	For hierarchical and complex designs, identification of the appropriate level for tests and full reporting of outcomes
☐	☒	Estimates of effect sizes (e.g. Cohen's *d*, Pearson's *r*), indicating how they were calculated

Our web collection on statistics for biologists contains articles on many of the points above.

Software and code

Policy information about availability of computer code

Data collection	Medoc thermode (ATS) and proprietary software were used during data collection.
Data analysis	Custom code in R, using R studio was used to analyse the data presented in this manuscript, as well as to create the figures. The code can be found here: https://github.com/Body-Pain-Perception-Lab/PHS-TemporalContrast. R packages used in this custom code are listed at the top of each script.

For manuscripts utilizing custom algorithms or software that are central to the research but not yet described in published literature, software must be made available to editors and reviewers. We strongly encourage code deposition in a community repository (e.g. GitHub). See the Nature Portfolio guidelines for submitting code & software for further information.

Data

Policy information about availability of data

All manuscripts must include a data availability statement. This statement should provide the following information, where applicable:

- Accession codes, unique identifiers, or web links for publicly available datasets
- A description of any restrictions on data availability
- For clinical datasets or third party data, please ensure that the statement adheres to our policy

The data are publicly available through GitHub on the above link and the Open Science Framework (https://osf.io/tp2q7/).

March 2021

Human research participants

Policy information about studies involving human research participants and Sex and Gender in Research.

Reporting on sex and gender	Gender information (male or female) were collected from each participant, with a final distribution of 121 females and 87 males. We included gender as a predictor in our model in the supplementary materials of the manuscript, no effect of gender on our main dependent variable was identified.
Population characteristics	Recruitment was fixed between 18-50 years of age, the mean age for our sample was 24.97 years (range 18-49, SD 5.19). Participants were recruited through participation pools at Aarhus University, Denmark and were therefore most likely educated and westernised individuals.
Recruitment	Participants were recruited through SONA Participation Pool at Aarhus University, Denmark.
Ethics oversight	The study protocal was approved by the local ethics commitee in Region MidtJylland, Denmark.

Note that full information on the approval of the study protocol must also be provided in the manuscript.

Field-specific reporting

Please select the one below that is the best fit for your research. If you are not sure, read the appropriate sections before making your selection.

☐ Life sciences ☒ Behavioural & social sciences ☐ Ecological, evolutionary & environmental sciences

For a reference copy of the document with all sections, see nature.com/documents/nr-reporting-summary-flat.pdf

Behavioural & social sciences study design

All studies must disclose on these points even when the disclosure is negative.

Study description	Quantitative experimental, mixed methods design
Research sample	Students and employees at Aarhus University, Denmark. The mean age for our sample was 24.97 years (range 18-49, SD 5.19). Out of 208 participants, 121 identified as female and 87 male. This sample is likely to be slightly higher educated, and contain a higher number of international, non-Danish participants than the Danish population. Participants were chosen through opportunity.
Sampling strategy	The sampling procedure was through convenience.
Data collection	Data were collected using a Medoc Thermal Probe, which was controlled using XXX software. Participants completed all conditions in one sitting and the innocuous condition was conducted prior to the noxious condition. The researcher was not blinded to the experimental conditions of the study.
Timing	August 2019 - March 2021
Data exclusions	7 participants were excluded from further analyses due to incomplete trial data.
Non-participation	0
Randomization	Participants were not allocated into experimental groups

Reporting for specific materials, systems and methods

We require information from authors about some types of materials, experimental systems and methods used in many studies. Here, indicate whether each material, system or method listed is relevant to your study. If you are not sure if a list item applies to your research, read the appropriate section before selecting a response.

Materials & experimental systems

n/a	Involved in the study
☒	☐ Antibodies
☒	☐ Eukaryotic cell lines
☒	☐ Palaeontology and archaeology
☒	☐ Animals and other organisms
☒	☐ Clinical data
☒	☐ Dual use research of concern

Methods

n/a	Involved in the study
☒	☐ ChIP-seq
☒	☐ Flow cytometry
☒	☐ MRI-based neuroimaging
